# Subjective Responses of Gynephilic Men and Women to Real versus Artificial Female Nudes

**DOI:** 10.1007/s10508-025-03357-2

**Published:** 2026-02-06

**Authors:** Ellen Zakreski, Alena Marečková, Ondřej Vaníček, Martin Hůla, Kateřina Klapilová, Jitka Lindová, James G. Pfaus

**Affiliations:** 1 Center for Sexual Health and Interventions, Czech National Institute of Mental Health, 25067 Klecany, Czech Republic; 2 Department of Psychology and Life Sciences, Faculty of Humanities, Charles University, Prague, Czech Republic

**Keywords:** Sexual attraction, Erotic pictures, Artificial intelligence, Computer-generated images, Hentai, Dolls

## Abstract

Depictions of visual sexual stimuli are changing with the advent of computer-generated imagery (CGI), hyper-realistic and idealized images created by artificial Intelligence (AI), and artistic styles such as hentai, that depict a diverse range of sexual situations and characteristics. Here we assessed the subjective realism, aesthetic value, subjective sexual attractiveness, and valence (pleasantness) of images depicting natural or surgically enhanced naked women taken from real photographs or in formats created by CGI, AI, or as dolls or hentai. Self-identified gynephilic males and females (*N* = 649) participated in a nationwide online survey about the perception of visual sexual stimuli. Although the real and AI-generated nudes were rated as significantly more realistic than the other categories, with the real images rated significantly more real than the others, AI-generated nudes were found more aesthetically appealing, sexually attractive, and pleasant than the other categories, with men rating nearly all categories higher than women (men and women found AI and CGI images equally aesthetically pleasing). There was a significant correlation of age, such that older participants found the real and AI-generated nudes more aesthetically appealing, sexually attractive, and pleasant than younger participants. In contrast, younger participants rated hentai significantly higher in these measures than older participants. These data suggest that AI-generated erotic material is superior to even real photographs in generating aesthetic appeal, positive valence, and ratings of sexual attractiveness, although both real and AI-generated nudes produced higher ratings in all measures compared to enhanced nudes, or those created by CGI, or as dolls or hentai.

## Introduction

Depictions of visual sexual stimuli (VSS) are as old as our species. From cave drawings to ancient pottery and “fertility” statues, to present-day pornographic images and videos presented in digital media, VSS are designed to stimulate fantasies and sexual arousal. But they also document the aesthetics of body types and sexual interactions, and how their positive and negative hedonic value differs across cultures and between epochs within a culture (Carl et al., 2012; Gabor, 1972; Koetzle & Scheid, 2014; Néret, 1995). With the advent of the internet and the current proliferation of digitized images and videos, along with artistic styles from different cultures, viewers now have access to a variety of images that depict many different types of sexual interactions and characters, from relatively realistic sexual interactions to fantasy-laden cartoons. This shift includes the addition of digital or digitally enhanced images, images of individuals with surgically enhanced secondary sex characteristics, life-like silicon sex dolls, and illustrated cartoons that allow for the depiction of more diverse sexual acts, body types, and age categories, including those that are atypical or not physically or legally possible (e.g., imaginary animals, unnatural body types, non-consensual sex).

Expectations of the content and quality of pornography have changed, along with ease of access, which has altered physiological and cognitive responses to VSS from different generations. For example, images that were highly arousing for young adults in the 1970s are only mildly arousing for subsequent generations of young adults (e.g., presence or absence of pubic and body hair; “natural” bodies versus airbrushed models with exaggerated secondary sex characteristics; D’Amours et al., 2013; Jacob et al., 2011). Thus, sexual images validated for research several decades ago, like the International Affective Picture System (IAPS; Lang et al., 1997), are not having the same effect they once did. This poses a serious challenge for ongoing research and the ability to replicate findings even using the same methodology and stimuli (e.g., Pfaus, 2023; Ziogas et al., 2020).

Attitudes toward a particular artistic style of erotic imagery or pornography may also vary between individuals depending on their exposure to that style (both with pornographic and non-pornographic media). Hentai is the pornographic form of a specific artistic style of cartoon (anime/manga), which originates from Japan and features highly exaggerated physical features (Cooper-Chen, 2012; Park et al., 2022) such as enlarged eyes and a triangular face and nose, along with fantasy-themed action depicting animals and super-human abilities. Anime/manga exists in various non-pornographic media (video games, comic books, television shows, films) targeting both child and adult audiences, and its popularity has spread to Western countries in the last few decades (Cooper-Chen, 2012; Pellitteri, 2021). In Western countries, pornography based on anime/manga may thus be particularly more appealing to younger generations, given that they have had greater exposure to this artistic style and are presumably more likely to have established connections to characters from stories drawn in this style.

Another challenge concerns the application of computer-generated imagery (CGI) and artificial intelligence (AI) to the creation or alteration of VSS (Alilunas, 2024; Lapointe & Dubé, 2024; Öhman, 2020; Saunders, 2019). Like hentai, CGI and AI can depict more diverse sexual acts and characters, and CGI, and especially AI, can create three-dimensional, realistic images. Although it is possible to surgically alter the appearance of real women, CGI and AI allow for more control in creating an idealized appearance. AI technology in particular appears to make ultra-realistic images (referred to as “deepfake”) with sharp visual resolution that older CGI technology cannot approximate. However, both can take a real photograph and alter the characteristics of an individual. In the case of VSS, this could include altering body shape, breast or pectoral proportion, genitalia, buttock size, facial features, hair color, eye color, and the sexual interactions of individuals. Although both technologies are used for human perception studies, to alter images of individuals or objects in advertising, and to make mainstream pornography, they can also be used for deception, exploitation, and fraud (where images of nonexistent individuals, or of real individuals doing something they did not do, including animation of deceased persons, are generated), and for the creation of illegal content (e.g., child pornography), through commercially available platforms (Becker & Laycock, 2023; Califano & Spence, 2024; Dobber et al., 2021; Döring et al., 2025; Hausken, 2024; Huang et al., 2021). CGI has also been used to create three-dimensional templates for life-like silicone sex dolls that have certain characteristics of “beauty” and sexual attractiveness desired by the purchaser.

An obvious question is how “real” these types of images are, e.g., to what extent people suspend disbelief in them as artificial or unrealistic. How might they compare to real persons or objects, not only in terms of perceived realism, but also in their subjective aesthetic value, sexual attractiveness, and overall pleasantness? The answer from the nascent literature seems to depend on the context and the willingness of individuals to suspend disbelief. For example, deepfakes can be used to create negative impressions of a politician, even if individuals viewing it know it is fake (Vaccari & Chadwick, 2020). In contrast, although AI-generated images of food are generally preferred over actual photographs of food, this effect is reduced if the viewer knows the AI images are fake (Califano & Spence, 2024). Faces made by both AI and CGI can be mistaken for real faces (Miller et al., 2023a, 2023b), although AI is superior to CGI in the appearance of realism. It is also the case that facial attractiveness ratings of real people are positively correlated with the attractiveness ratings of CGI faces and that both adults and children prefer attractive faces presented as social partner avatars (Principe & Langlois, 2013).

With the advent of surgery, CGI, AI, sex dolls, and the popularity of artistic styles such as hentai, super-realistic sexual imagery is now widely accessible. It can be difficult for viewers to know whether they are viewing real or artificial imagery, particularly as it relates to AI and some CGI, in addition to images of surgically enhanced secondary sex characteristics. It is not clear whether people find these images to be as appealing or sexually attractive as images of people who have not undergone surgical (or digital) augmentation. Additionally, it is not clear to what extent viewers consider the former categories to be realistic. Given the potential of CGI or AI to create pornographic images or to enhance particular anatomical aspects of real individuals (e.g., body shape and facial features), the presumed appeal of hentai images in pornographic websites, and the assumed attractive aesthetics of silicone sex doll features, the present exploratory study examined how static images of naked women taken from real photographs, or in formats created by AI, CGI, real photographs of women with surgically exaggerated body parts, or as sex dolls or hentai, are rated for subjective realism, sexual attractiveness, aesthetic appeal, and valence (unpleasantness to pleasantness), by gynephilic men and women.

We conducted an online nationwide self-report survey in the Czech Republic about exposure to erotic visual depictions of naked women. This included questions regarding sociodemographic information, sexual orientation and interest, sexual experience with female partners, and exposure to naked female imagery, and asked participants to rate the realism, aesthetic value, sexual attractiveness, and valence of different versions of female nude figures.

### Participants

A final sample of 649 participants (604 self-identified men, 45 self-identified women) was included from a total of 760 respondents. Recruitment advertisements invited Czech-speaking adults aged 18 and older who are attracted to women to participate in a nationwide online survey on perceptions of sexual images of women, with an opportunity to enter a lottery for one of ten 1000 CZK (approximately $43 USD) prizes. Note that the Czech word for woman "žena" also means female. Participants were recruited through an online volunteer platform for sexuality research (sexlabnudz.cz) and social media advertisements. The platform, which is managed by the Czech National Institute of Mental Health, allows Czech-speaking adults from the general public to register in a database for participation in studies on sexual health. Additionally, individuals engaged in outreach programs, such as a project focused on paraphilic interests (Project Paraphile, parafilik.cz), were invited to join the platform. As a result, the sample may over-represent individuals with paraphilic interests. Upon registering in this database, individuals provided basic demographic information, which allowed for targeted recruitment. For the present study, only participants who had indicated a gynephilic sexual orientation (based on the Kinsey scale) were emailed the recruitment advertisement. We therefore contacted bisexual individuals, homosexual women and heterosexual men. Note that attraction to women was also assessed in the survey itself (see below). Recruitment advertisements were also posted on the platform’s website and its associated social media page (facebook.com/sexlabnudz/about).

Participants that were included ranged in age from 18 to 91 (*M* = 34.45 years, *SD* = 11.68). Age was negatively skewed. Since skewness was 1.07, which is between − 2 and 2, it is acceptable for ANOVA and regression (Hair et al., 2017). To be eligible for the study, participants had to be older than 18 years and be sexually attracted to adult women. Participants were asked to rate their sexual attraction to women on a 6-point Likert scale ranging from zero (definitely not) to five (definitely yes). Participants were excluded if they scored below four (*n* = 55). Included participants could thus be bisexual adults, homosexual women, or heterosexual men. Participants were also excluded if they reported high levels of sexual interest in minors using items described in Zakreski et al. (2024). Briefly, participants were asked to rate 1) how sexually aroused they are by the idea of sexual contact with a child without signs of adolescence (up to 12 years), and 2) how sexually aroused they are by the idea of sexual contact with a pubescent girl/boy (a child already with signs of adolescence between the ages of 12 and 15). For each of these two items, participants rated how aroused they were on a five-point Likert scale ranging from one (definitely not) to five (definitely yes). Participants scoring above three on either item were excluded (*n* = 53) so that they could be referred to a similar ongoing study focusing on minor attracted individuals. As mentioned earlier, the initial pool of participants likely had a disproportionately high number of individuals with paraphilic interests, since individuals who participated in our center's support program for paraphilia were recommended to join the database of volunteers which received recruitment ads. The sample thus includes people without a strong attraction to minors. Finally, three participants did not identify as either man or woman. Given the small size of this group and our plan to include gender as a predictor in our statistical models, this group was excluded from the analyses. Of the original 760 participants, 649 were analyzed.

To determine the minimum sample size, a power analysis was conducted with effect size partial eta-squared (η_p_^2^) = .01 (small effect) for six stimulus categories of naked women (see below), for α = .05, power = .95, and effect size f = 0.1 (converted from η_p_^2^ of .01). This analysis generated a minimum sample size of 166.

### Stimuli

**Fig. 1:**
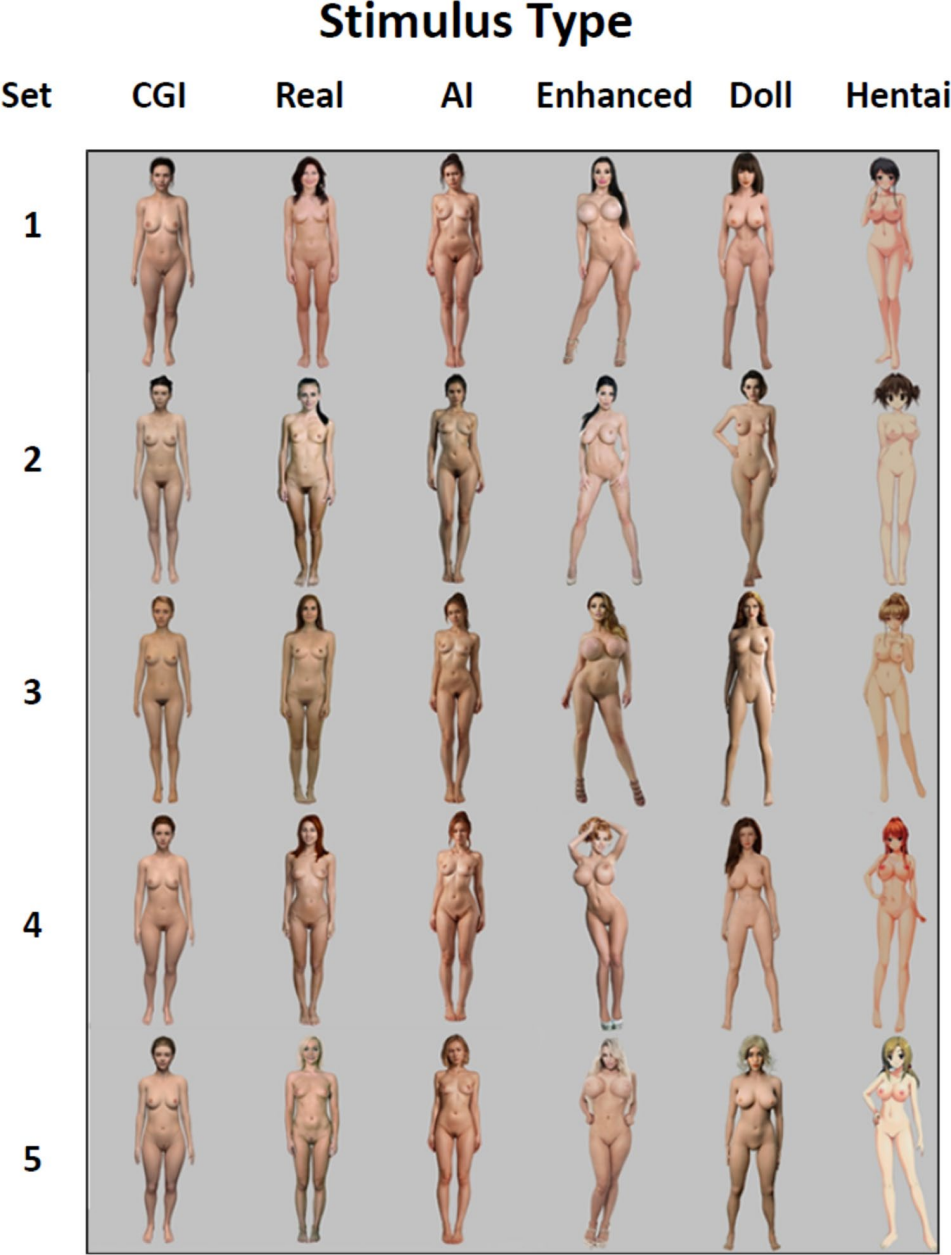
Stimuli used in the present study

Participants were exposed to six categories of static stimuli depicting naked female figures against a gray background (Fig. 1): (1) CGI-generated female figures (CGI), (2) photographs of real women (Real), (3) AI-generated women (AI), (4) real women with surgically enhanced lips, breasts, and hips (Enhanced), (5) silicone sex dolls (Dolls), and (6) hentai. Each category contained five female figures with different characteristics (hair color (black, brown, blond, dark blond, red) and body type (voluptuous, athletic, or petite), to account for the fact that different individuals are attracted to different body types and hair colors). While other factors (ethnicity, height, facial features) may influence attractiveness, we did not consider variation in other characteristics as testing individual reactions to all combinations of multiple characteristics would significantly increase the burden on participants and it would also make it more difficult to find stimuli. Figures had lighter skin tones to align with the ethnic composition of the Czech population, where approximately 89.8% are ethnically Czech (Czech Statistical Office, 2024).

Premade templates of female figures with different body shapes and hair colors were purchased from the set of available avatars at DAZ 3D Studio (DAZ3D.com), a company that sells 3D templates of characters and objects for graphic arts. These premade templates were then refined in collaboration with 3dsense (3dsense.cz), to create five CGI naked adult females with distinct body types and hair colors: a voluptuous black-haired figure with larger breasts, an athletic brown-haired figure, an athletic dark blond figure, a red-haired figure with rounded hips, and a petite blond figure. CGI figures had waist-to-hip ratios of approximately 0.7. Breasts were designed to appear more natural as opposed to having a surgically enhanced appearance. For instance, each breast was smaller than the face, breasts were not spherical, and did not protrude from the chest at a 90-degree angle. Each figure had a slightly different skin tone, and some had pubic hair. All pictures depicted the figure in the middle of the picture from a frontal view, standing with feet slightly apart, hands mostly along the body, a neutral expression, hair tied in a ponytail and on a gray background.

For the remaining stimulus categories, the second author sought images that resembled the appearance of each CGI female figure from pictures of real women, real women with surgically enhanced lips, breasts and hips, hentai, and sex dolls. The selection included at least three pictures for corresponding figures in each stimulus category. The second author (AM) and two male researchers (OV, MH) then rated the pictures as to which were most similar to the CGI-generated figures. For the pictures of real women, we searched the ALS scan database (alsscan.com), from Budapest 2019, Czech 2019, and Czech 2022 castings, with permission. We cannot verify whether these women received plastic surgery or not, but they were chosen to resemble the CGI figures which were designed to have a relatively natural appearance as described above. The pictures of real women with apparent surgically enhanced body parts and hentai were searched on available sites using Google with the safe-search function turned off. For the enhanced group, we sought images of women who appeared to have surgically enhanced, exaggerated sexual characteristics, such as very low waist-to-hip and waist-to-bust ratios, and spherical breasts, where the nipple faced upward at a 90-degree angle or more from the chest, and large lips that had puffier corners and a more uniform shape compared to the CGI avatar. We searched for these images using search terms synonymous with large or extra-large breasts, hips, lips and the term, plastic surgery as well as specific hair colors. We did not verify that any of the enhanced women in the images had plastic surgery. We used pictures of silicone sex dolls listed in the shop Naughty Harbour (naughtyharbor.cz) after obtaining their permission. An online AI-powered platform, nudify.art, was used to generate three AI figures that resembled each female figure using several text prompts (Table 1), and the best figures were selected by agreement of AM, OV, and MH.

**Table 1:** Text prompts used for the present data set

***prompt Blue*** Positive: young woman, ((full body including legs and feet, full figure:1.3)), distance, nude, naked, black hair, ponytail, small smile, frontal view, standing still, standing straight, straight pose, straight posture, arms close to body, legs close to each other, full gray background, tiny tits, (small breasts) Negative: ((big breasts)), visible teeth, walk, walking
***prompt Alana*** Positive: teenage woman, attractive face, ((full body including legs and feet)), (distance), nude, naked, black hair, long hair, smiling, front, standing still, standing straight, arms close to body, full gray background, tiny tits, big breasts, thick thighs Negative: ((small breasts, tiny tits)), visible teeth, walk, walking
***prompt Magda*** Positive: teenage woman, attractive face, ((full body including feet, full figure:1.3)), nude, naked, ginger hair, long hair in a bun, front, standing still, standing straight, straight pose, straight posture, arms close to body, legs close to each other, full gray background, ((tiny tits, small breasts)), narrow thighs, narrow pelvis, narrow shoulders, thin waist Negative: ((big breasts)), visible teeth, walk, walking
***prompt Salvia*** Positive: teenage woman, attractive face, ((full body including feet, full figure:1.3)), nude, naked, blonde hair, short hair, front, standing still, standing straight, straight pose, straight posture, arms close to body, legs close to each other, full gray background, ((tiny tits, small breasts)), narrow thighs, narrow pelvis, narrow shoulders Negative: ((big breasts)), visible teeth, walk, walking
***prompt Rowena*** Positive: teenage woman, ((full body including feet, full figure:1.3)), nude, naked, red head, red hair, (long hair in a bun), front, standing still, standing straight, straight pose, straight posture, arms close to body, legs close to each other, full gray background, thick thighs, wide pelvis, narrow shoulders Negative: ((small breasts)), visible teeth, walk, walking
***For change of pubic hair*** Positive: trimmed, narrow trim, thin strip, small, tiny Negative: –

Engine: https://nudify.art/

Date: September 23th 2023

After the final selection of female figures (five figures for each of the six stimulus categories), the images were then edited in Adobe Photoshop, in which the background was unified to the same gray color for all pictures and in which we removed tattoos and jewelry. We unified the skin tone of all female figures in each category according to that of the initial CGI-generated figure using Color Sampler to edit the RGB channel (Photoshop Training Channel, 2022) across categories. We also adjusted the pubic hair of real women to match it with the CGI-generated figures. We did not alter the pubic hair of sex dolls and hentai figures, which are normally presented without pubic hair. The enhanced images also lacked pubic hair. It was also necessary to slightly change the hair color of several images to better match the corresponding figure. Stimuli were presented at a resolution of 300 × 450 pixels. Several differences remained which could not be adjusted without significantly disrupting the figures. Specifically, most female figures had their hair down and not in a ponytail, as in the CGI figure. Since we had to search for preexisting images for the real, enhanced, doll and hentai categories, there were some differences in terms of posture and facial expression. While most of the figures had a neutral expression, some figures in the real, enhanced, and hentai categories were smiling. Additionally, the surgically enhanced real women were all wearing high heels and their pose and expression were therefore potentially more seductive. Some of the doll and hentai figures were also in different poses (some had one hand placed on the hip or under the chin, and some had both hands behind the back).

### Measures

Participants’ sociodemographic information, including their age, sex, education level, and information about their sexual orientation, relationship status, number of sex partners, sex and masturbation frequency, paraphilic preferences, porn use and experience with each stimulus category, was collected.

**Fig. 2:**
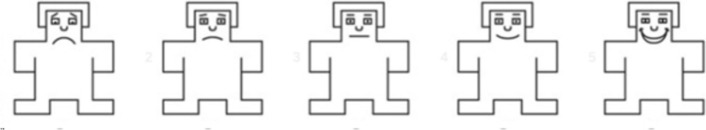
Response options for the Self-Assessment Manikin scale, from 1 (left) to 5 (right)

Subjective ratings of realism (Do jaké míry na Vás žena působí realisticky? To what extent does this woman seem realistic to you?), sexual attraction (Nakolik Vás žena sexuálně přitahuje? How sexually attractive is this woman to you?), and aesthetic appeal (Jak se Vám žena líbí po estetické stránce? How do you like this woman in terms of aesthetics?) were made for each picture in each category. We used a slider with values from 0 to 100, where 0 represented the lowest rating (female figure does not look real, is not sexually attractive, and is not aesthetically appealing), whereas 100 represented the highest rating (female figure looks very real, is very sexually attractive, and is very aesthetically appealing). The slider was automatically set to 0 at the beginning of each rating. We also assessed valence (pleasantness) using the Self-Assessment Manikin scale (SAM; Lang, 1980). The SAM is a self-report tool developed to measure features of an emotional response (Bradley & Lang, 1994) using a cartoon-like manikin. In this study, a 5-point pictorial scale was utilized for valence/pleasantness of the image, ranging from one (unpleasant) to five (pleasant), which are shown in Fig. 2. Specifically, participants were instructed that the scale represents feelings of negativity/positivity, and to choose the one on the left if the image made them feel “unhappy, annoyed, dissatisfied, melancholic, desperate, or bored," or to choose the one on the right if the image made them feel "happy, pleased, satisfied, hopeful, or relaxed” (Tato škála představuje pocity negativity/pozitivity. Pokud jste se během sledování cítil/a “nešťastně, otráveně, nespokojeně, melancholicky, zoufale, znuděně”, zaškrtněte panáčka zcela vlevo. Pokud jste se cítil/a “šťastně, potěšeně, spokojeně, nadějeplně, uvolněně”, zaškrtněte panáčka zcela vpravo).

### Procedure

The survey was generated and hosted via the online platform Qualtrics (qualtrics.com). Participants’ written informed consent was obtained through the online survey system, before they were able to answer additional questions. Participants were informed that they could withdraw from the study at any time. Further, we also offered the opportunity to participate in a competition in the form of a lottery for a financial reward for ten randomly selected winners (1000 CZK, approximately $43 USD). In order to enter the competition, participants had to leave their email at the end of the questionnaire. Data were collected from October to December 2023.

**Fig. 3:**
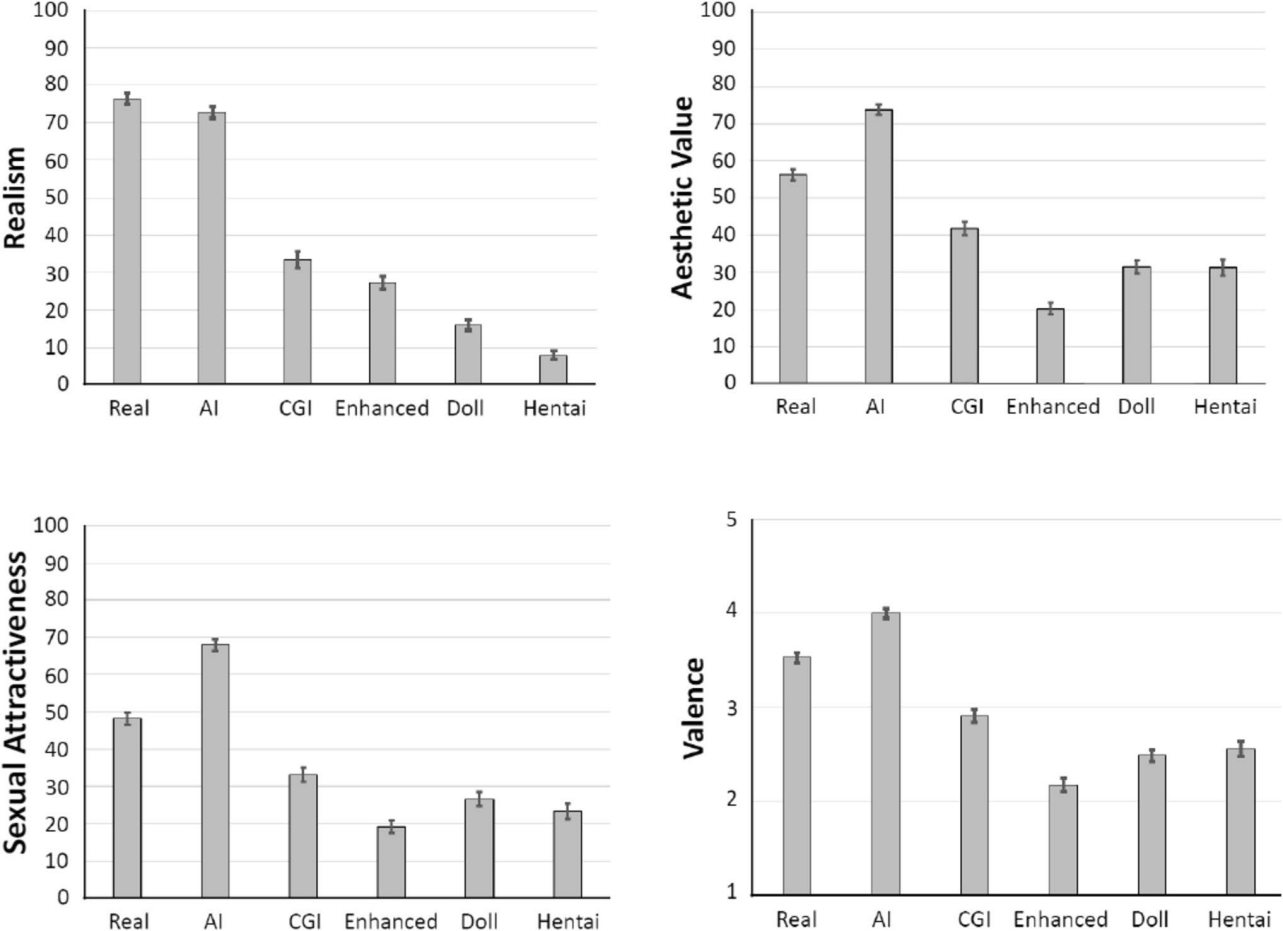
Mean realism, aesthetic value, sexual arousal, and valence ratings for each stimulus category summed across gender and age. Top Left: Mean realism ratings ± SEM. Mean ratings of each stimulus category were significantly different from one another. Top Right: Mean aesthetic value ratings ± SEM. Mean ratings of each stimulus category were significantly different from one another. Bottom Left: Mean sexual attractiveness ratings ± SEM. Mean ratings were significantly different, except the hentai category, which did not differ significantly from the sex doll or enhanced category. Bottom Right: Mean valence ratings ± SEM. All stimulus categories were significantly different, except the doll and hentai categories, which did not differ significantly from each other

The survey consisted of three blocks in this order: (1) sociodemographic questions and questions concerning sexual orientation, paraphilic interests, sexual behaviors, and pornography use, (2) ratings of static pictures of naked female Fig. 3 ratings of dynamic videos (data not included here).

The order of static image presentation was randomized for each participant. Each trial began with the presentation of one image along with four subjective ratings (sexual attractiveness, aesthetic appeal, realism, and valence). Each participant was presented with all 30 images (five female figures x six stimulus categories), one at a time. The median completion time for the entire survey was 32.4 min.

### Statistical Analyses

Statistical analyses were performed using RStudio (version 2021.09.2, Build 382). First, for each of the four subjective ratings, for each participant, we averaged the scores on that subjective rating across the five female figures for that stimulus category. Consequently, for each of the four subjective ratings, each participant had six scores (one for each stimulus category). To examine the effect of stimulus on each subjective rating, we created four mixed ANOVAs. The dependent variable was the subjective rating (sexual attractiveness, aesthetic appeal, realism, and valence), the within-subjects factor was stimulus (real, AI, CGI, enhanced, doll, hentai), and the between-subject independent variables were gender and age (which was mean centered). Greenhouse–Geisser corrections were applied for each ANOVA, since Mauchly's test indicated that the sphericity assumption was violated. ANOVAs (in addition to detection of and correction for sphericity violations) were performed using the “afex” package (Singmann et al., 2024). Post hoc tests (including tests of simple main effects) were performed using the “emmeans” package (Lenth, 2022). The Bonferroni–Holm method was used to adjust post hoc *p*-values for multiple comparisons. Given that valence was rated on a five-point Likert scale, which may not necessarily reflect an interval scale, in addition to the mixed ANOVA, we performed a Friedman within-subjects nonparametric test to examine the effect of stimulus category on valence, followed by Bonferroni–Holm-adjusted Wilcoxon post hoc tests.

## Results

### Prior Sexual Experience and Exposure to Naked Female Imagery

Of the final sample of 649 participants, 601 (92.60%) reported having had at least one female sexual partner in their lifetime. When asked if they had ever watched pornography, 633 (97.69%) responded yes, 15 (2.31%) responded no, and 1 person chose not to answer. Among the participants that reported watching pornography at least once, Table 2 shows how many have either never, sometimes, or often encountered pornography belonging to each stimulus category. Only 4.74% of participants reported no exposure to images of real people, while a much larger percentage reported no exposure to AI-generated pornography (60.19%), or no exposure to pornography involving sex dolls (65.72%).

**Table 2:** Frequency of exposure to different categories of pornography among participants who have watched pornography

Stimulus category	Never	Sometimes	Often
Visual representation of real people	30/633 (4.74%)	119/633 (18.80%)	484/633 (76.46%)
AI-generated	381/633 (60.19%)	228/633 (36.02%)	24/633 (3.79%)
Computer-generated (CG)	268/633 (42.34%)	313/633 (49.45%)	52/633 (8.21%)
Surgically enhanced (enlarged breasts, hips, lips)	81/633 (12.80%)	349/633 (55.13%)	203/633 (32.07%)
Doll (silicone doll)	416/633 (65.72%)	204/633 (32.23%)	13/633 (2.05%)
Hentai	185/633 (29.23%)	366/633 (57.82%)	82/633 (12.95%)

### Effect of Stimulus Category on Perceived Realism

A mixed ANOVA was performed to determine the effect of stimulus category (real, AI, CGI, enhanced, doll, or hentai) on ratings of realism. The ANOVA revealed a large and significant main effect of stimulus, *F*(3.12, 2013.91) = 498.90, η_p_^2^ = .436, *p* < .001. Figure 3 (top left) shows how realism ratings differed between the six stimulus categories. Post hoc tests (with Bonferroni–Holm-adjusted *p*-values) revealed that realism differed significantly between all stimuli (all |*t*|≥ 2.25, all adjusted *p* ≤ .025). The most realistic category was real, followed by AI, CGI, enhanced, doll, and hentai.

**Fig. 4:**
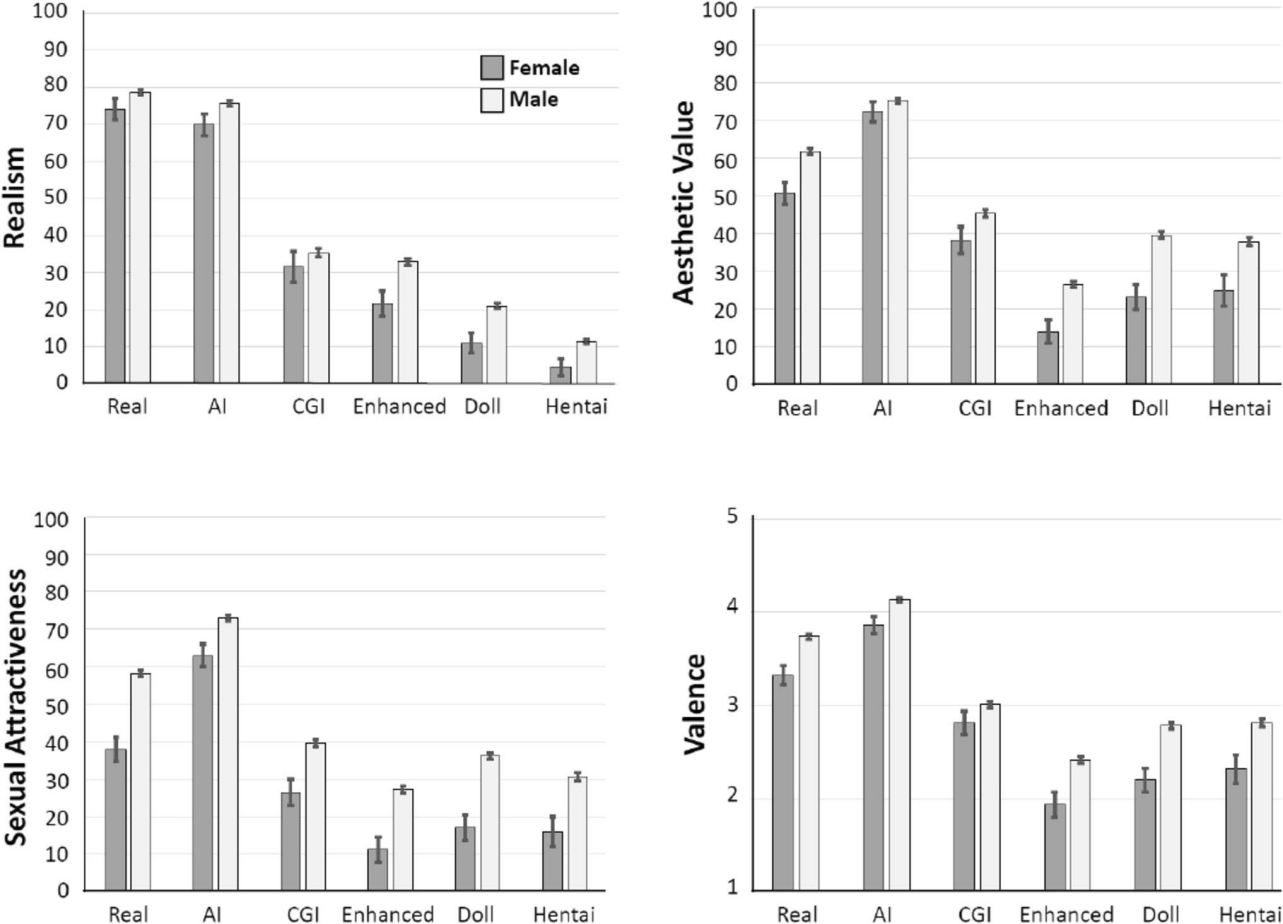
Mean realism, aesthetic value, sexual arousal, and valence ratings for each stimulus category by gender. Top Left: Mean realism ratings ± SEM. Overall, all stimulus categories were rated significantly higher by males than females. Top Right: Mean aesthetic value ratings ± SEM. Ratings for the AI and CGI categories did not differ between males and females, whereas ratings of the other stimulus categories differed significantly. Bottom Left: Mean sexual attractiveness ratings ± SEM. Ratings for all stimulus categories were significantly higher for males than females. Bottom Right: Mean valence ratings ± SEM. Males rated the valence for all stimulus categories as significantly higher (more pleasant) than females

Figure 4 (top left) shows realism ratings made by females and males across the six stimulus categories. The ANOVA detected a significant main effect of gender, *F*(1, 646) = 10.60, η_p_^2^ = .016, *p* = .001, with females rating the images overall as less realistic than males. The interaction between gender and stimulus was not significant, *F*(3.12, 2013.91) = 1.34, η_p_^2^ = .002, *p* = .260.

**Fig. 5:**
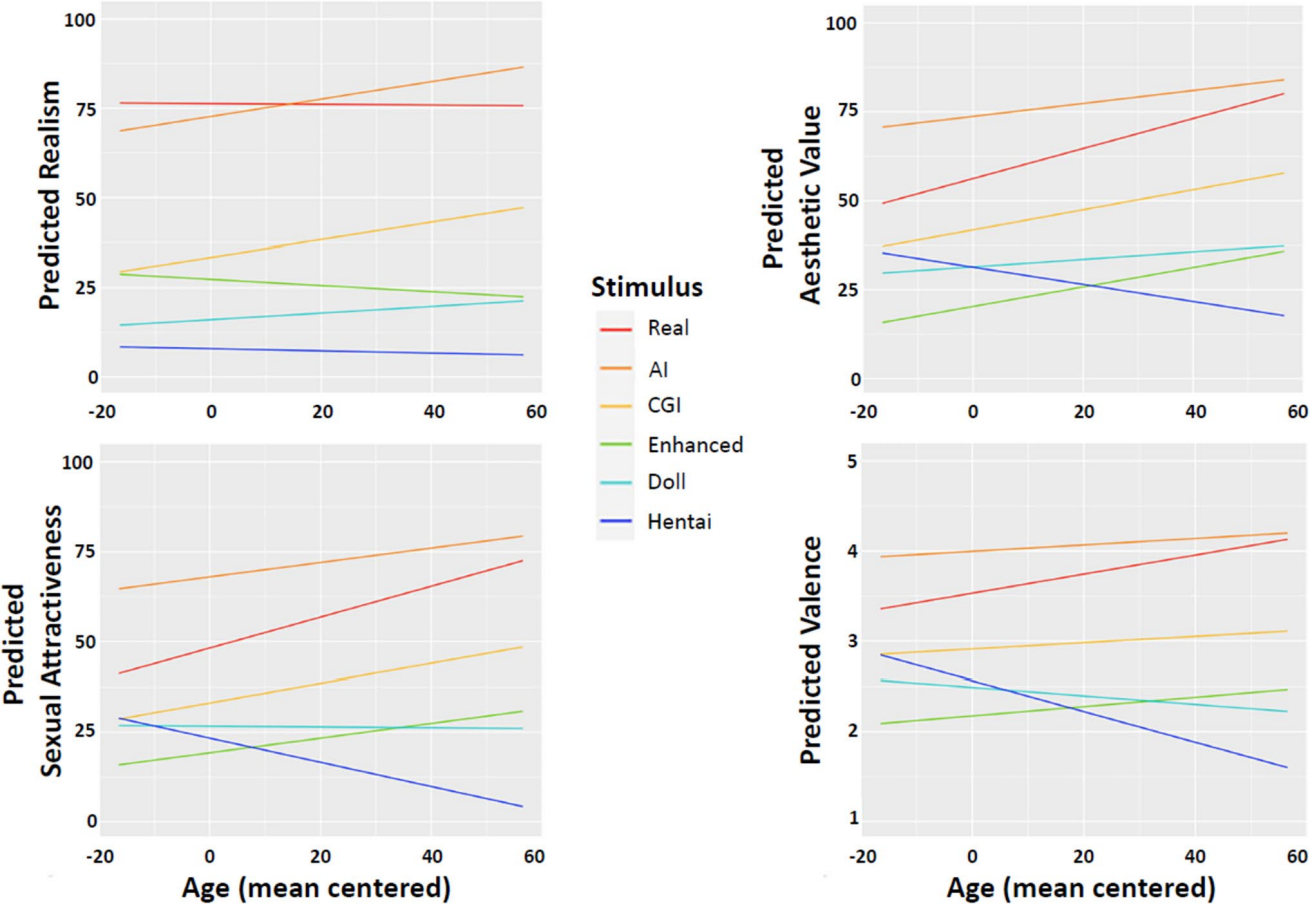
Predicted realism (top left), aesthetic value (top right), sexual attractiveness (bottom left), and valence (bottom right) across mean centered age for each stimulus category. Flat lines indicate a similar rating across age. Lines with a positive slope indicate higher ratings by older compared to younger participants. Lines with a negative slope indicate higher ratings by younger compared to older participants

There was no significant main effect of age on overall realism ratings, *F*(1, 646) = 2.59, η_p_^2^ = .004, *p* = .108. However, there was a significant interaction between age and stimulus category, *F*(3.12, 2013.91) = 6.34, η_p_^2^ = .01, *p* < .001. To explore this interaction, tests of simple main effects were performed to examine the linear effect of age on realism ratings within each stimulus category. As shown in Fig. 5 (top left), the relationship between age and realism ratings differed between categories. Older individuals rated the AI category as significantly more realistic relative to younger individuals (*β* = 0.24, *t* = 3.64, *r* = .14, adjusted *p* = .002). Realism ratings for the CGI category also correlated positively and significantly with age (*β* = 0.24, *t* = 2.62, *r* = .10, adjusted *p* = .046). In contrast, realism ratings for the remaining four categories did not change significantly with age (all |*t*|≤ 1.51, |*r*|≤ .06, unadjusted *p* ≥ .131).

### Effect of Stimulus Category on Aesthetic Value

The mixed ANOVA found a significant main effect of stimulus on ratings of aesthetic value, *F*(3.77, 2434.30) = 233.28, η_p_^2^ = .265, *p* < .001. Figure 3 (top right) shows the aesthetic value for each of six stimulus categories. Post hoc tests revealed that aesthetic value ratings differed significantly between all stimuli (all |*t*|≥ 4.76, adjusted *p* < .001), except ratings for the doll and hentai categories did not differ significantly from one another (*t* = 0.04, unadjusted *p* = .972). The stimulus category rated as most aesthetically pleasing was AI, followed by real, CGI, and doll (which did not differ from hentai), while the enhanced category was the least aesthetically pleasing.

The ANOVA detected a significant main effect of gender, *F*(1, 646) = 17.50, η_p_^2^ = .026, *p* < .001, such that males found the images significantly more aesthetically pleasing than females (Fig. 4, top right). There was also a small but significant interaction between gender and stimulus, *F*(3.77, 2434.30) = 3.47, η_p_^2^ = .005, *p* = .009. Although post hoc tests showed that differences between stimulus categories followed the same pattern for males and females, the ratings were lower among females compared to males, but only significantly lower compared to males for the real, enhanced, doll, and hentai categories (all *t* ≥ 3.04, adjusted *p* ≤ .007). Aesthetic value ratings did not differ significantly between males and females for the AI category or CGI category (all *t* ≤ 1.94, adjusted *p* ≥ .106).

The ANOVA also revealed a significant main effect of age, *F*(1, 646) = 9.86, η_p_^2^ = .015, *p* = .002, with older individuals rating the images overall as more aesthetically pleasing. A significant interaction of age and stimulus was found, *F*(3.77, 2434.30) = 16.71, η_p_^2^ = .025, *p* < .001 (Fig. 5, top right). Tests of simple main effects showed that older individuals found the real, AI, CGI, and enhanced images more aesthetically pleasing than younger individuals (all *t* ≥ 3.11, *r* ≥ .12, adjusted *p* ≤ .006). In contrast, the aesthetic value of the doll category did not vary significantly with age, *β* = 0.11, *t* =  − 1.41, *r* = .06, unadjusted *p* = .158. Older individuals found the hentai category less aesthetically pleasing than younger individuals (*β* =  − 0.24, *t* =  − 2.62, *r* =  − 10, adjusted *p* = .018).

### Effect of Stimulus Category on Sexual Attractiveness

The mixed ANOVA found a significant overall main effect of stimulus on sexual attractiveness, *F*(3.80, 2454.56) = 190.58, η_p_^2^ = .228, *p* < .001. Figure 3 (bottom left) shows the ratings of sexual attractiveness for each of the stimulus categories. According to post hoc tests, sexual attractiveness ratings differed significantly between all stimuli (all |t|≥ 4.24, adjusted *p* < .001), except the hentai category did not differ significantly from the enhanced (*t* = 1.94, unadjusted *p* = .057), or doll categories (*t* =  − 1.88, unadjusted *p* = .061). The AI images were rated as most sexually attractive, followed by real, CGI, doll, hentai, and then enhanced (although hentai did not differ from doll and enhanced).

The ANOVA detected a significant main effect of gender, *F*(1, 646) = 36.27, η_p_^2^ = .053, *p* < .001, with females rating the images as less sexually attractive than males (Fig. 4, bottom left). The interaction between gender and stimulus showed a weak trend, *F*(3.80, 2454.56) = 2.05, η_p_^2^ = .003, *p* = .088.

The ANOVA further revealed a small significant main effect of age, *F*(1, 646) = 5.05, η_p_^2^ = .008, *p* = .025, such that sexual attraction scores increased with age overall. However, the ANOVA also detected a significant interaction of age and stimulus category, *F*(3.80, 2454.56) = 21.00, η_p_^2^ = .031, *p* < .001. Figure 5 (bottom left) shows the effect of age on sexual attractiveness ratings for each stimulus category. Similar to what was found for aesthetic value ratings, tests of simple main effects showed that older individuals found the real, AI, CGI, and enhanced categories significantly more sexually attractive than younger individuals (all *t* ≥ 2.70, *r* ≥ .11, adjusted *p* ≤ .014). In contrast, the sexual attractiveness of the doll category did not vary significantly with age (β =  − 0.01, *t* =  − 0.14, *r* = -.005, unadjusted *p* = .890). Similar to aesthetic value, older individuals found the hentai category significantly less attractive than younger individuals (β =  − 0.34, *t* =  − 3.78, *r* =  − 15, adjusted *p* < .001).

### Effect of Stimulus Category on Valence

The ANOVA revealed a significant overall main effect of stimulus on ratings of valence, *F*(3.87, 2500.91) = 184.81, η_p_^2^ = .222, *p* < .001. Figure 3 (bottom right) shows the valence for each stimulus category. Post hoc tests revealed that valence ratings differed significantly between all stimuli (all |*t*|≥ 4.22, adjusted *p* < .001), except ratings for the doll and hentai categories did not differ significantly from each other (*t* =  − 1.05, unadjusted *p* = .295). Valence ratings were highest for AI, followed by real, CGI, then hentai (which did not differ significantly from the doll), with the enhanced category receiving the lowest ratings. Mean scores for the real and AI categories were above three (95% confidence intervals (CIs) = 3.43–3.64, and 3.90–4.09, respectively) indicating a somewhat positive valence (pleasantness). The mean valence for the CGI category was 2.91 (95% CI = 2.78–3.04), indicating a somewhat neutral valence, while the mean valences for the enhanced, doll, and hentai categories were ≤ 2.57 (95% CI = 2.04–2.31, 2.36–2.62, 2.41–2.72), indicating a somewhat negative valence (unpleasantness). Again, given that valence was rated on a five-point Likert scale, which may not necessarily reflect an interval scale, the Friedman test was used to examine the effect of stimulus category on valence (Friedman's test), followed by Bonferroni–Holm-adjusted Wilcoxon sign-rank post hoc tests. The results of the nonparametric analysis were consistent with the previous findings, revealing a significant main effect of stimulus, χ^2^(5) = 1666.72,* p* < .001, Kendall’s W = .514. Nonparametric post hoc tests showed the same results, with all stimulus categories differing significantly from one another, except the doll and hentai categories.

The ANOVA also detected a significant main effect of gender, *F*(1, 646) = 20.23, η_p_^2^ = .030, *p* < .001. Females rated the images overall as less pleasant than males (Fig. 4, bottom right). As with sexual attractiveness, the interaction between gender and stimulus showed a weak trend, *F*(3.87, 2500.91) = 2.04, η_p_^2^ = .003, *p* = .089.

There was no significant main effect of age on valence ratings, *F*(1, 646) = 0.01, η_p_^2^ < .001, *p* = .925. However, the ANOVA detected a significant interaction of age and stimulus,

*F*(3.87, 2500.91) = 19.29, η_p_^2^ = .029, *p* < .001. As can be seen in Fig. 5 (bottom right), valence scores generally increased with age for the more realistic categories, while for the two least realistic categories (doll and hentai), age was inversely associated with valence. Simple main effect tests were conducted to examine the effect of age on valence for each stimulus category. Older individuals rated the real image category as significantly more pleasant relative to younger individuals (*β* = 0.01, *t* = 4.61, *r* = .18, adjusted *p* < .001). There was also a positive association between age and valence for the AI, enhanced, and CGI categories, but these associations were weak and non-significant (all *t* ≤ 1.70, *r* ≤ .07, unadjusted *p* ≥ .09). Younger individuals found the hentai category significantly more pleasant (β =  − 0.02, *t* = -4.88, *r* =  − 19, adjusted *p* < .001) than older individuals. There was also an inverse association between age and valence for the doll category, but this was not significant (β =  − 005, *t* =  − 1.64, *r* =  − 06, unadjusted *p* = .101).

## Discussion

This study compared two-dimensional still images of naked females taken from CGI female figures, photographs of real women, AI-generated female figures, real women with surgically enhanced lips, breasts, and hips, sex dolls, and hentai, along subjective ratings of realism, aesthetic value, sexual attractiveness, and valence (pleasant or unpleasant) made by gynephilic men and women. While AI-generated nudes were rated as significantly less realistic overall than real images, they were regarded as significantly more realistic than the other categories. However, the AI-generated nudes were found significantly more aesthetically appealing, sexually attractive, and pleasant, than the other categories (including real images). Men generally rated all categories higher than women along these dimensions, although the overall pattern for women was the same as men. The aesthetic value of the AI and CGI images did not differ significantly between genders. There was also a significant correlation of age, such that older participants found the CGI and AI-generated images more realistic, and the real and AI-generated nudes more aesthetically appealing, sexually attractive, and pleasant than younger participants, whereas younger participants rated hentai significantly higher in aesthetic value, sexual attractiveness, and pleasantness compared to older participants. To our knowledge, this study is the first of its kind to compare real versus artificial erotic stimuli along these dimensions, and the results have important implications for the study of subjective and objective sexual arousal and erotic aesthetics, especially with the new applications of AI-generated imagery.

It would appear that, similar to still images of food (e.g., Califano & Spence, 2024) and faces (Miller et al., 2023b), still images of female nudes generated by AI are superior to real pictures, in this case along dimensions of aesthetic value, sexual attractiveness, and pleasantness. This may be due to the sharp visual resolution of the images, despite the fact that, in this study, the AI-generated images were perceived as less real. In all assessments, CGI were midway between AI and real, whereas the enhanced, doll, and hentai images were rated lower, and in the unpleasant range of the valence. However, this latter effect seemed to be driven by older participants, as the younger ones rated the hentai as significantly more aesthetically pleasing, sexually attractive, and pleasant than the older individuals. In almost all cases, the patterns across different ages for the image categories shown in Fig. 5 were similar for aesthetic value, sexual attractiveness and valence, but differed from perceived realism in all but the AI and CGI categories. Interestingly, the images of the dolls had the same positive association with age for realism and aesthetic value, but an inverse association with age for sexual attractiveness and valence. Although this could indicate that realism is not a strong predictor of aesthetic value, sexual attractiveness, and positive or negative valence of a naked female image (especially for younger individuals), it was the case here that real and AI images had the highest overall ratings in all measures, and maintained that pattern across both gender and age.

It is not clear why gynephilic women found many of the images less real, aesthetically appealing, sexually attractive, and of lower (less pleasant) valence than the males. Heterosexual women in the Czech Republic view erotic and pornographic material similarly to men, and without apparent gender-related differences in stimulus content (Krejčova et al., 2023). We assume the same for gynephilic women. Previous studies have reported that both men and women show stronger arousal responses for attractive versus unattractive real images, especially if they depict their preferred versus non-preferred gender (Timmers et al., 2025). A recent study using both implicit association and priming tasks showed that gynephilic women had a strong cognitive bias toward images of females compared to androphilic women who did not show a bias toward images of females or males (Snowden et al., 2024). Thus, we would have expected the gynephilic women in our sample to have similar responses to men, which they did regarding stimulus categories overall, but with less robust ratings. It is possible that gynephilic women have different ideas of what constitutes a realistic or attractive female body compared to gynephilic men. Indeed, one study found that similar to gynephilic men, self-identified lesbians and bisexual women prefer a 0.7 waist-to-hip ratio and larger breasts, but not necessarily a thin body type (Cohen & Tannenbaum, 2001). Another study found that gynephilic women preferred a potential romantic partner with a higher percentage of body fat compared to heterosexual males (Cordes et al., 2021). It is important to note that many of the female figures used here had a thin appearance.

The images rated as less real (surgically enhanced, sex doll, and hentai) had the lowest ratings across all other measures (and were rated lower by women compared to men), and a downward slope pattern across age (except realism ratings were non-significantly positively correlated with age for the doll category). This is interesting, especially in terms of the images of surgically enhanced women, which appear to defy evolutionary psychological notions about enhanced secondary sex characteristics as inherently attractive (e.g., because they confer a perception of greater fertility or maternal resources; Buss & Schmitt, 2019). The attractiveness of surgically enhanced individuals may thus be an acquired preference (e.g., Pfaus et al., 2020). Similarly, the hentai images were rated significantly greater in aesthetic value, sexual attractiveness, and valence by younger participants compared to older participants. Pornhub began publishing data regarding popular download search terms in 2013. That year, hentai was not among the top 20 most popular search terms worldwide (Pornhub, 2013). In 2015, hentai was the 11th most popular search term worldwide (Pornhub, 2015). From 2017 to 2019, it became the second most common (Pornhub, 2017, 2018, 2019). Since 2021, hentai is now the most common search term (Pornhub, 2021, 2022, 2023). While anime/manga are relatively novel in the western world (Cooper-Chen, 2012; Park et al., 2022; Pellitteri, 2021), hentai as a genre of VSS is not new. While the artistic style of hentai has changed, it can trace its roots to Shunga, a form of erotic art in Japan during the 16th Century that evolved into erotic manga by the 19th Century. The term “hentai” means “perverse” in Japanese, and hentai cartoons that depicted “abnormal” fetish and paraphilic sexual themes began to appear during the middle of the Meiji era in Japan (late 19th to early 20th Century; Carl et al., 2012). These have continued to evolve to the present day. Hentai has been referred to as a form of sexual exploitation material (SEM; Steel et al., 2021), and it was reported to be among the more diverse categories of SEM that convicted users of child SEM download (Steel et al., 2021). However, hentai has also become popular among millennials and Gen Zs (e.g., Loule, 2020), and the Pornhub data suggest that downloading hentai imagery from sexually explicit websites is something done more by younger compared to older individuals (Pornhub, 2023). The present study suggests this is true of younger individuals in the Czech Republic as well.

The hentai phenomenon discussed above is an example of generational differences in physical attributes found positive, aesthetically appealing, and sexually attractive. Another potential difference is in the enhanced category across age, which showed the opposite effect of being rated higher in valence by older relative to younger participants. Older individuals in our sample likely came into their sexual debut viewing both natural women and those with surgically enhanced features in erotic and pornographic magazines. This is also reminiscent of the generational differences in the preference for pubic hair. Preferences for female models with pubic hair have decreased over the past 50 years, consistent with the depiction of females with less and less pubic hair in mainstream erotic and pornographic material (Herbenick et al., 2010; Ramsey et al., 2009; Schick et al., 2011). The removal of pubic hair by women in Western cultures has generally followed this trend, with more younger individuals opting for complete genital depilation compared to older individuals (Butler et al., 2015; Sangiorgi et al., 2017). As with hentai, surgically enhanced features, and genital depilation, it is possible that early experience viewing or fantasizing about particular sexually arousing characteristics comes to imprint a preference for those characteristics which is subsequently observed as a generational difference in preference, valence, attractiveness, etc. The implications of this are problematic for the generation of validated sets of erotic images or videos (e.g., Prantner et al., 2024; Wierzba et al., 2015) that can be used as stimuli in studies of cognitive and/or physiological sexual arousal across different generations (D’Amours et al., 2013; Jacob et al., 2011). Indeed, cultural differences in preferred stimuli also exist (Rowland and Uribe, 2020). However, AI has the potential to create erotic images of the same individuals that would vary by preferred generational or cultural features, thus allowing different generational and/or cultural preferences to be examined explicitly. It would also be of interest in future studies to examine male nudes using the same categories for androphilic men and women.

### Conclusions and Limitations

The present study found that AI-generated images of naked females had greater aesthetic value, were more sexually attractive, and generated a more pleasant valence than real photographs, CGI, surgically enhanced images, dolls, or hentai depicting naked women, in a cross-sectional study of gynephilic men and women in the Czech Republic. Limitations of the study included a smaller sample of women compared to men, the lack of objective or subjective measures of sexual arousal, and limited measures of realism, aesthetics, sexual attractiveness, and valence. Additionally, the sample is not representative of the general population due to the recruitment strategy, which targeted individuals registered on a sexuality research volunteer platform, followers of the research group’s social media page, and participants in clinical outreach programs for paraphilic interests (see above). This likely resulted in an over-representation of individuals with an interest in sexuality research or paraphilic interests (although we excluded individuals with high levels of pedophilic or hebephilic interests). Furthermore, the nude images had relatively similar ethnic features and ages. Future research should attempt to replicate this study using non-white figures and figures of other ages. Another limitation is the difficulty in maintaining consistency of appearance across stimulus categories. For instance, hair style, pose, and the presence of pubic hair and shoes varied between stimulus categories. Nevertheless, the findings of this study provide one of the first comparisons of the impact of real versus CGI/AI-generated or enhanced erotic images. Future studies should examine similar images of real and artificial males by androphilic women and men, extend the image range to other types of VSS, especially those that depict different ethnicities, and to depictions of sexual interactions, including fetish and paraphilic behaviors. It would also be important to examine differences in preference within other cultures.

## Data Availability

Data and statistical analysis code can be obtained by contacting the first author (EZ).
